# Characterization of Fosfomycin Resistant Extended-Spectrum β-Lactamase-Producing *Escherichia coli* Isolates from Human and Pig in Taiwan

**DOI:** 10.1371/journal.pone.0135864

**Published:** 2015-08-17

**Authors:** Sung-Pin Tseng, Sheng-Fan Wang, Cheng-Yu Kuo, Jun-Wei Huang, Wei-Chun Hung, Guan-Ming Ke, Po-Liang Lu

**Affiliations:** 1 Department of Medical Laboratory Science and Biotechnology, College of Health Sciences, Kaohsiung Medical University, Kaohsiung, Taiwan, ROC; 2 Department of Marine Biotechnology and Resources, National Sun Yat-sen University, Kaohsiung, Taiwan, ROC; 3 Division of Infectious Diseases, Kaohsiung Medical University Chung-Ho Memorial hospital, Kaohsiung, Taiwan, ROC; 4 Department of Internal Medicine, Ministry of Health and Welfare Pingtung Hospital, Kaohsiung, Taiwan, ROC; 5 Department of Microbiology and Immunology, Kaohsiung Medical University, Kaohsiung, Taiwan, ROC; 6 Graduate Institute of Animal Vaccine Technology, National Pingtung University of Science and Technology, Neipu, Pingtung, Taiwan, ROC; 7 Department of Laboratory Medicine, Kaohsiung Medical University Hospital, Kaohsiung, Taiwan, ROC; 8 College of Medicine, Kaohsiung Medical University, Kaohsiung, Taiwan, ROC; 9 Department of Internal Medicine, Kaohsiung Medical University Hospital, Taiwan, Kaohsiung, ROC; Ross University School of Veterinary Medicine, SAINT KITTS AND NEVIS

## Abstract

To investigate the efficacy of fosfomycin against extended-spectrum β-lactamases (ESBL) producing *Escherichia coli* in Taiwan and the resistance mechanisms and characterization of human and pig isolates, we analyzed 145 ESBL-producing isolates collected from two hospitals (n = 123) and five farms (n = 22) in Taiwan from February to May, 2013. Antimicrobial susceptibilities were determined. Clonal relatedness was determined by PFGE and multi-locus sequence typing. ESBLs, *ampC*, and fosfomycin resistant genes were detected by PCR, and their flanking regions were determined by PCR mapping and sequencing. The fosfomycin resistant mechanisms, including modification of the antibiotic target (MurA), functionless transporters (GlpT and UhpT) and their regulating genes such as *uhpA*, *cyaA*, and *ptsI*, and antibiotic inactivation by enzymes (FosA and FosC), were examined. The size and replicon type of plasmids carrying fosfomycin resistant genes were analyzed. Our results revealed the susceptibility rates of fosfomycin were 94% for human ESBL-producing *E*. *coli* isolates and 77% for pig isolates. The PFGE analysis revealed 79 pulsotypes. No pulsotype was found existing in both human and pig isolates. Three pulsotypes were distributed among isolates from two hospitals. IS*Ecp1* carrying *bla*_CTX-M-group 9_ was the predominant transposable elements of the ESBL genes. Among the thirteen fosfomycin resistant isolates, functionless transporters were identified in 9 isolates. Three isolates contained novel amino acid substitutions (Asn67Ile, Phe151Ser and Trp164Ser, Val146Ala and His159Tyr, respectively) in MurA (the target of fosfomycin). Four isolates had fosfomycin modified enzyme (*fosA3*) in their plasmids. The *fosA3* gene was harboured in an IncN-type plasmid (101 kbp) in the three pig isolates and an IncB/O-type plasmid (113 kbp) in the human isolate. In conclusion, we identified that 6% and 23% of the ESBL-producing *E*. *coli* from human and pigs were resistant to fosfomycin, respectively, in Taiwan. No clonal spread was found between human and pig isolates. Functionless transporters were the major cause of fosfomycin resistance, and the *fosA3*-transferring plasmid between isolates warrants further monitoring.

## Introduction

The emerging problem of extended-spectrum β-lactamases (ESBLs) producing *Enterobacteriaceae* has been reported worldwide [1,2]. Infections of ESBLs-producing *Enterobacteriaceae* are difficult to treat because many of the commonly used antibiotics (β-lactams) will not work against these isolates. Falagas et al. reported that fosfomycin could be a solution to this problem; the susceptibility to fosfomycin was 96.8% (1604/1657) in ESBL-producing *Escherichia coli* and 81.3% (608/748) in *Klebsiella pneumoniae* in a systemic review [3]. In addition, oral fosfomycin use is effective against uncomplicated urinary tract infections caused by ESBL-producing *E*. *coli*, *Proteus mirabilis*, *K*. *pneumoniae* and *Staphylococcus saprophyticus* [3–6].

The fosfomycin resistance mechanisms that have been reported include the modification of the antibiotic target (MurA), functionless transporters (GlpT and UhpT transporters and their regulating genes such as *uhpA*, *cyaA*, and *ptsI*) and antibiotic inactivation (FosA and FosC) in *E*. *coli* [7]. Due to the fosfomycin inactivation genes (*fosA3* and *fosC*) that were found in the conjugative plasmid or transposon element, transferable *fosA3* and *fosC* genes have become a serious problem in East Asia [8–14]. Recent studies have focused on the disseminative fosfomycin resistant genes. These reports revealed *fosA3* genes were flanked by IS*26* and were localized on conjugative plasmids for human isolates in Japan [13,14], Korea [12], and another *fosA* variant (*fosKP96*) was found in Hong Kong [8], while some studies have focused on livestock or animal isolates from different regions of China [9–11]. In this study, we aimed to identify fosfomycin resistance and its underlying mechanisms and characterization of ESBL-producing *E*. *coli* isolates from human and pig sources in Taiwan.

## Materials and Methods

### Bacterial isolates

A total of 145 ESBL-producing isolates of *E*. *coli* were collected from 2 hospitals and 5 local farms in Taiwan during February-May 2013. Ninety-five isolates were collected from the Kaohsiung Medical University Hospital (KMUH), and the sources included urine (n = 69), blood (n = 7), abscess (n = 8), and others (n = 11). Twenty-eight isolates were collected from the Pingtung Hospital (PTH), and the sources included urine (n = 18), blood (n = 6), pus (n = 3), and sputum (n = 1). Twenty-two pig isolates were all collected from fecal specimens from pigs. The sizes of farms were ranged from 2000 to10000 feeder pigs (Landrace and American Yorkshire pigs). The body weight and age ranged from 7 to 25 Kg and 28 Days to 8 weeks, respectively. All pig isolates were belonging to fecal sample isolated from anal swabs. The distances from hospitals to pig farms were about 20 kilometers.We analysed the bacterial isolates only and the categories of human specimen information or the private information of patient were not included in the study.

### Ethics statement

This study was approved by the Institutional Review Board (IRB) of Kaohsiung Medical University Chung-Ho Memorial Hospital, Kaohsiung, Taiwan (KMUH-IRB-860-911). The study subjects were bacterial isolates and the written consent given by the patients was waived by the approving IRB. Before collecting fecal specimens from pigs on this study, we contacted the farm owners and obtained their permission. No specific permits were required for the described field studies and the locations where we sampled are not privately-owned or protected in any way. The field studies did not involve endangered or protected species.

### Antimicrobial susceptibility testing

The antimicrobial susceptibility testing was performed by a standard agar dilution method according to the guidelines of the Clinical and Laboratory Standards Institute (CLSI) [15]. Three antimicrobial agents: fosfomycin, cefotaxime, and meropenem were tested in all isolates. The phenotypic detection of ESBLs was conducted by Vitek 2 system (bioMérieux Inc., Durham, NC). The susceptibilities of 4 *fosA3*-habouring isolates and their transformants were tested for the following antimicrobial agents including ampicillin, amikacin, tetracycline, levofloxacin, chloramphenicol, fosfomycin, cefotaxime, and meropenem.

### Bacterial strain typing (PFGE and MLST)

The PFGE typing of XbaI (New England BioLabs, Ipswich, MA)-digested DNA was prepared in accordance with previously described methods [16]. Dice similarity indices were employed to construct the dendrogram of the pulsotype relationships through the unweighted pair group method using arithmetic averages (UPGMA) with BioNumerics software version 6.5 (Applied Maths). The pulsotypes were assigned to the same clusters if they exhibited 80% similarity in the dendrogram. The MLST scheme of *E*. *coli* uses internal fragments from seven housekeeping genes: *adk* (adenylate kinase), *fumC* (fumarate hydratase), *gyrB* (DNA gyrase), *icd* (isocitrate/isopropylmalate dehydrogenase), *mdh* (malate dehydrogenase), *purA* (adenylosuccinate dehydrogenase), and *recA* (ATP/GTP binding motif). The primers were derived from the *E*. *coli* MLST database (http://mlst.ucc.ie/mlst/dbs/Ecoli). The PCR amplification and sequencing were performed following this website’s suggested protocols. The four *fosA3*-habouring isolates were analyzed by MLST.

### Detection of antimicrobial resistance genes

The plasmid DNA was extracted using the QIAGEN Plasmid Mini Kit. For the detection of the ESBLs (*bla*_SHV_, *bla*_TEM_, *bla*_CTX-M-group 1_, *bla*_CTX-M-group 2_, and *bla*_CTX-M-group 9_) and plasmid-mediated AmpC genes *bla*_DHA_, and *bla*_CMY_ and the fosfomycin resistance genes (*fosA*, *fosA3*, *fosC2*), the primer sets from previous reports were used [10,17]. The purified PCR amplicons of *bla*_TEM_ were sequenced by the dideoxy chain-termination method with an automated DNA Sequencer (Perkin-Elmer ABI3700), and the nucleotide sequences were analyzed with the BLAST sequence alignment database (National Center for Biotechnology Information).

### Analysis of the transposable elements upstream of the *bla* genes

To investigate the different kinds of transposable elements surrounding the *bla*SHV, *bla*CTX and *bla*TEM genes, the linkage of *bla*SHV with *recF* and IS*26* was determined using specific primers (RECF-F and SHV-F-I; IS26-FCJ and SHV-F-I) [18]. The linkage of *bla*TEM with *tnpA* and IS*26* was determined using specific primers (RH401 and RH410; RH1270 and RH606) [19]. The linkage of *bla*CTX-M-group 1 with IS*Ecp1* and IS*26* was determined using specific primers (ISEcpUP and CTX-M1RCJ; IS26-FCJ and CTX-M1RCJ) [18]. The linkage of *bla*CTX-M-group 9 with IS*Ecp1* was determined using specific primers (ISEcpUP and CTX-M-9-R1) [18]. All the PCR amplicons were sequenced, and the sequence was compared in the GenBank nucleotide database.

### Nucleotide sequencing of *fosA3* and the flanking regions

To determine the genetic environment of *fosA3*, PCR mapping and an LA PCR *in vitro* cloning kit (Takara Shuzo Co. Ltd., Japan) were used. The flanking regions between IS*26* and *fosA3* were identified by PCR amplicons and sequencing. The primers used were those from previous studies [9].

### Conjugation and transformation assays

The conjugation and electotransformation assays were performed as previous studies [12]. The conjugation experiments were conducted according to the broth mating method using *E*. *coli* J53 as the recipient. The transconjugants were selected on LB agar plates supplemented with sodium azide (150 μg/ml), fosfomycin (40 μg/ml), and glucose-6-phosphate (G6P) (25 μg/ml). If the conjugation assay failed, an electotransformation assay using *E*. *coli* DH5α as the recipient was conducted. The transconjugants were selected on LB agar plates supplemented with fosfomycin (40 μg/ml) and G6P (25 μg/ml).

### Plasmid analysis and Southern blot hybridization

The plasmid incompatibility group analyses were determined according to the PCR-based replicon-typing method [20]. The size estimation of the plasmids was determined by an S1-nuclease PFGE analysis and calculated with the BioNumerics GelCompar software package (Version 5.0, Applied Mathematics, Sint-Martens-Latem, Belgium) as previously described [17]. The Lambda Ladder PFG marker (New England Biolabs, Ipswich, MA, USA) was used as a molecular size marker. The plasmid PFGE gel was then subjected to Southern blotting and hybridized with a DIG (digoxigenin)-labeled *fosA3* and CTX-M-group 9 specific probe. The primer sets from previous reports were used [10,17]. The hybridization assay was performed as described previously [17].

### Activity of GlpT and UhpT transpoters

The usage of carbohydrates which indicated the activity of GlpT and UhpT transpoters was performed as previous studies [21,22]. Briefly, 0.2% (w/v) G6P or *sn*-glycerol 3-phosphate (G3P) as the sole carbon source was supplied in M9 minimal medium agar. The overnight bacterial suspension was washed with an equivalent volume of saline and optimal McFarland No. 4. The bacterial suspensions (200 μl) were plated on M9 minimal medium agar supplying different sole carbohydrates at 37°C for 48 h. The negative phenotype was defined as a lack of colonies forming on the plate.

### Sequencing analysis of fosfomycin resistance related chromosomal genes

PCR and sequencing were performed to amplify the entire sequences of the 6 genes including *murA*, *glpT*, *uhpT*, *uhpA*, *ptsI*, and *cyaA*. The primers used were those from a previous study [22]. The variations of amino acids were compared to the reference strain *E*.*coli* ATCC 25922.

### Cloning overexpression of MurA from the different amino acid variations

The *murA* gene (ATCC25922, A12, A19, and K55) was amplified by PCR using a cloning primer, as previously described [22]. The PCR products were cloned into a T & A (Yeastern Biotech, Taiwan) plasmid and were transformed into *E*. *coli* DH5α. The insertion of the *murA* gene was verified by nucleotide sequence. To determine the fosfomycin susceptibility of the overexpressing *murA* in *E*. *coli*, 25 μg/ml G6P and 1 mM isopropyl β-D-1-thiogalactopyranoside (IPTG) were added to Muller-Hinton agar with various concentrations of fosfomycin (0–1024 μg/ml).

## Results

### Antimicrobial susceptibility tests and molecular typing

**Fig 1:**
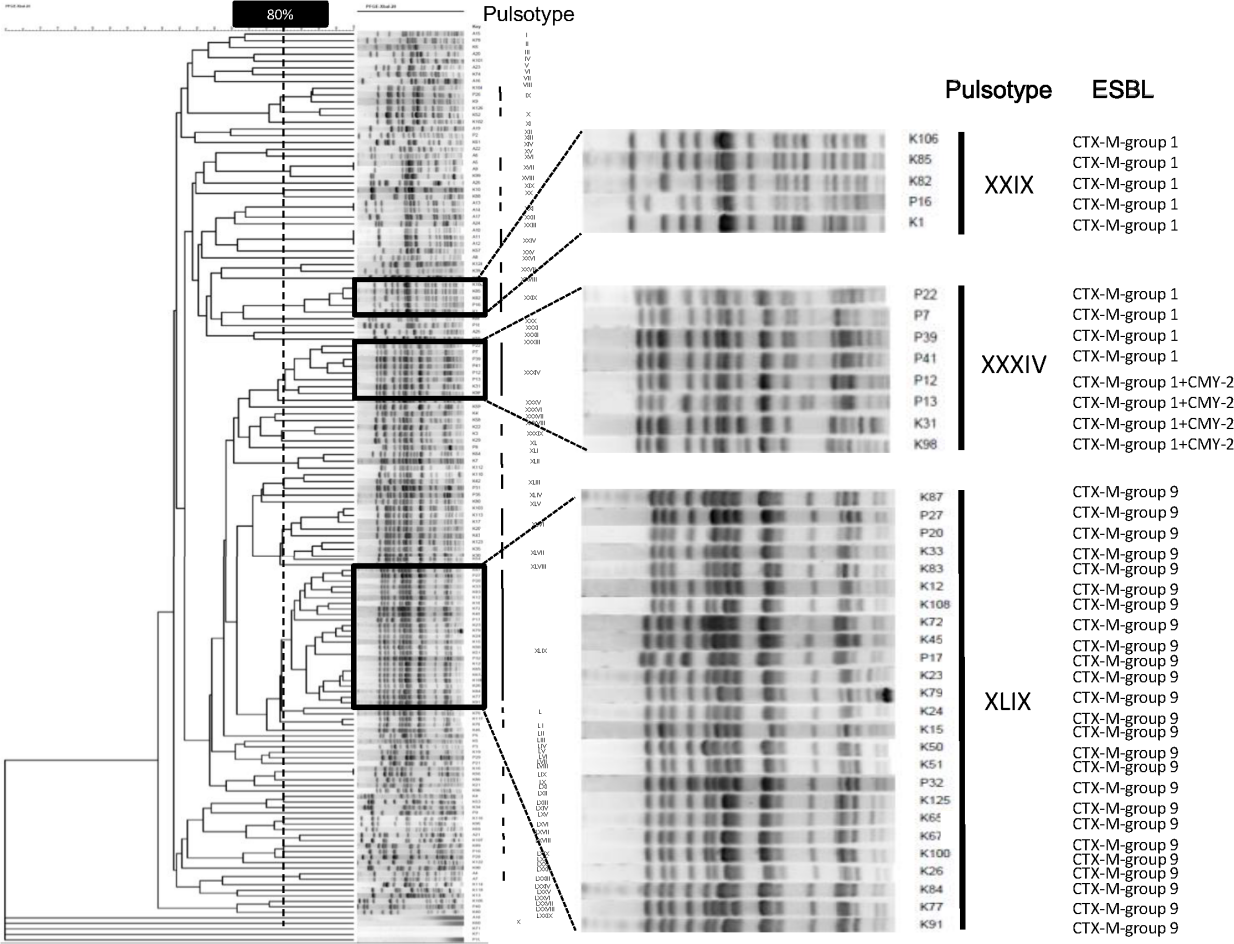
A dendrogram of pulsotype relationships developed via the unweighted pair group method using arithmetic averages (UPGMA) with BioNumerics software version 6.5 (Applied Maths). Pulsotypes were assigned to the same clusters if they exhibited 80% similarity in the dendrogram. Three major clusters (XXIX, XXXIV, and XLIV) were found in two hospitals.

The susceptibility rates for fosfomycin were 94% (90/95), 89% (25/28), and 77% (17/22) in KMUH, PTH and pig ESBL-producing *E*. *coli* isolates, respectively. The PFGE analysis revealed 79 pulsotypes and 4 non-typable isolates, indicating the genetic diversity of these isolates (Fig 1). Three pulsotypes appeared in both hospitals, including pulsotype XXIX (n = 5, 4 from KMUH and 1 from PTH), XXXIV (n = 8, 2 from KMUH and 6 from PTH) and XLIV (n = 25, 21 from KMUH and 4 from PTH). CTX-M-group 1 was dominant ESBL in Pulsotype XXIX and XXXIV whereas CTX-M-group 9 was dominant ESBL in puslotype XLIX, respectively. No identical puslotypes were found in both human and pig isolates; diversity of puslotypes were found in pig isolates. This finding indicates that human ESBL-producing *E*. *coli* has no relationship with pig isolates.

### Detection of β-lactamases and their linkage to transposable elements

Among the 95 isolates in KMUH, the most frequent β-lactamases were CTX-M-group 9 (n = 36; 37%), followed by TEM (n = 24; 25%), CMY-2 (n = 21; 22%), and the CTX-M-group 1 (n = 18; 18%) (Table 1). Similarly, for the 28 isolates from PTH, the CTX-M-group 1 was the most common type (n = 10; 35%), followed by TEM (n = 9; 32%), CMY-2 (n = 9; 32%), and CTX-M-group 9 (n = 7; 25%). For the pig isolates, the CTX-M-group 9 gene was the dominant type (n = 14; 63%), followed by TEM (n = 12; 54%), the CTX-M-group 1 (n = 6; 27%), and CMY-2 (n = 5; 22%). These data found that the CTX-M-group 1, the CTX-M-group 9 and CMY-2 were the most frequent ESBLs in Taiwan. Besides, it should be noted that all TEM were TEM-1 which was not ESBL in this study.

**Table 1:** Antibiotic resistant genes of the ESBL-producing *E*. *coli* human and pig isolates.

Antibiotic resistance genes	No. (%) of total isolates in KMU hospital (n = 95)	No. (%) of total isolates in PT hospital (n = 28)	Pig isolates from 5 farms (n = 22)
**β-lactamases**			
SHV	8 (8)	1 (3)	0 (0)
TEM-1^a^	24 (25)	9 (32)	12 (54)
CTX-M-group 1	18 (18)	10 (35)	6 (27)
CTX-M-group 2	0 (0)	0 (0)	0 (0)
CTX-M-group 9	36 (37)	7 (25)	14 (63)
DHA-1	3 (3)	0 (0)	0 (0)
CMY-2	21(22)	9 (32)	5 (22)
TEM-1+ CTX-M-group 1	8 (8)	1 (4)	1 (5)
TEM-1+ CTX-M-group 9	5 (5)	4 (14)	7 (32)
TEM-1+ CMY-2	5 (5)	6 (21)	2 (10)
TEM-1+ CTX-M-group 1+CMY-2	1 (1)	0 (0)	1 (5)
TEM-1+ CTX-M-group 9+CMY-2	2 (2)	0 (0)	1 (5)
TEM-1+ CTX-M-group 1+DHA-1	1 (1)	0 (0)	0 (0)
SHV+TEM-1+CMY-2	3 (3)	0 (0)	0 (0)
**Fosfomycin metallo-enzyme**			
*fosA*	0 (0)	0 (0)	0 (0)
*fosA3*	1 (1)	0 (0)	3 (13)
*fosC2*	0 (0)	0 (0)	0 (0)

a: TEM-1 was determined by sequencing.

Transposable elements were usually found in the upstream region of ESBLs and could be related to horizontal genetic transferring [18,19]. PCR mapping and sequencing revealed different transposable elements related to ESBLs (S1 Fig). In the upstream region of SHV, two types were found: *recF*- *kdpC*- *bla*_SHV_ (SHV_Type A_) and IS*26*- 73 bp spacer sequence- *bla*_SHV_ (SHV_Type B_). IS*Ecp1* was found upstream of the CTX-M-group 1 gene, which was the dominant type (83% of the KMUH isolates, 90% of the PTH isolates, and 50% of the pig isolates) (S1 Table). Two different lengths of spacer regions (42 bp in CTX-M-G9_Type A_ and 127 bp in CTX-M-G9_Type B_) were found between IS*Ecp1* and *bla*_CTX-M-group 9_. CTX-M-G9_Type A_ was the predominant type in the two hospitals and in the pig isolates.

### Characteristics of fosfomycin-resistant *E*. *coli* isolates

Among the 145 ESBL-producing *E*. *coli* isolates, 13 isolates (9.0%) were resistant to fosfomycin (Table 2). Four and five isolates grew on minimal medium agar supplemented with G3P and G6P, respectively (Table 2). To determine the resistance mechanism, nucleotide sequences of the genes *glpT*, *uhpT*, *uhpA*, *ptsI* and *cyaA* were tested in all the resistant isolates. Among the 9 isolates that did not grow on G3P, four isolates (A19, K66, K116 and P12) revealed amino acid substitutions in the *glpT* gene. The A19 and K66 isolates were identified to have single amino acid substitutions at codons 174 (Leu174Val) and 209 (Arg209His), respectively. K66 isolate also carried *fosA3* gene that causes fosfomycin resistance. The K116 isolate had 2 amino acid changes (Gly142Cys and Phe176Ser), and the P12 isolate had 3 amino acid changes (Arg50Cys, Ala156Val, and Val149Met). Two isolates (K55 and K101) which did not grow on G3P did not have amino acid changes in the *glpT* gene but possessed amino acid substitutions in the upstream regulator *ptsI* (Thr215Ala and Gly227Arg in K55 and Ala104Thr in K101). Three isolates (A10, A11, and A12) did not have amino acid changes in the *glpT* and *ptsI* genes; however, these isolates contained the *fosA3* gene that results in fosfomycin resistance.

**Table 2:** Characteristics of fosfomycin-resistant *E*. *coli* isolates and reference strains.

Isolate No.	Source	Pulsotype	ESBL type	*fosA3*	MIC FOS		Growth		Amino acid substitution		
					G6P(+)	G6P(-)	G3P	G6P	MurA	GlpT	UhpT
**ATCC25922**					1	2	+	+	None	None	None
**A5**	Pig farm	XVII	CTX-M-group 9		128	256	+	-	None	None	None
**A10**	Pig farm	XXIV	CTX-M-group 9	*+*	512	1024	-	+	None	None	Ser122Ile Trp151Cys Phe187Tyr
**A11**	Pig farm	XXIV	CTX-M-group 9	*+*	256	512	-	+	None	None	None
**A12**	Pig farm	XXIV	CTX-M-group 9	*+*	256	1024	-	+	Asn67Ile	None	Arg83Cys
**A19**	Pig farm	XII	CTX-M-group 9		512	1024	-	-	Phe151Ser Trp164Ser	Leu174Val	None
**K55**	KMUH	XXXIII	CMY-2		256	512	-	-	Val146Ala His159Tyr	None	Val85Leu
**K66**	KMUH	XXX	CTX-M-group 9 +CMY-2	*+*	256	1024	-	+	None	Arg209His	None
**K72**	KMUH	XLIX	CTX-M-group 9		128	256	+	-	None	Arg50Cys Ala156Val	Ser26Arg His50Pro Ile149Met
**K101**	KMUH	V	SHV+ CMY-2		128	256	-	-	None	None	Cys109Trp
**K116**	KMUH	LXVI	CTX-M-group 9 + CMY-2		128	256	-	-	None	Gly142Cys Phe176Ser	Val18Leu
**P12**	PTH	XXXIV	CTX-M-group 1 +CMY-2		128	256	-	+	None	Arg50Cys Ala156Val Val149Met	Lys132Glu Val143Met Tyr165His
**P16**	PTH	XXIX	CTX-M-group 1		128	256	+	-	None	None	Lys132Glu Ile149Met Tyr165His
**P32**	PTH	XLIX	CTX-M-group 9		128	256	+	-	None	Arg50Cys Ala156Val	Trp44Cys Gly134Asp

Among the 8 isolates that did not grow on G6P, 6 isolates (K55, K72, K101, K116, P16 and P32) revealed amino acid substitutions in the *uhpT* gene (Table 2). The K55, K101 and K116 isolates were identified to have single amino acid substitutions at codons 85 (Val85Leu), 109 (Cys109Trp) and 18 (Val18Leu), respectively. Multiple amino acid substitutions were found in K72 (Ser26Arg, His50Pro and Ile149Met), P16 (Lys132Glu, Ile149Met and Tyr165His), and P32 (Trp44Cys and Gly134Asp). Although A5 and A19 did not grow on G6P and did not have any amino acid changes in the *uhpT* gene, the upstream regulator *uhpA* contained two amino acid substitutions (Pro160Leu and Ala167Val in A5; Gln32His and Ala39Val in A19).

### Characterization of 4 *fosA3*-habouring isolates

**Fig 2:**
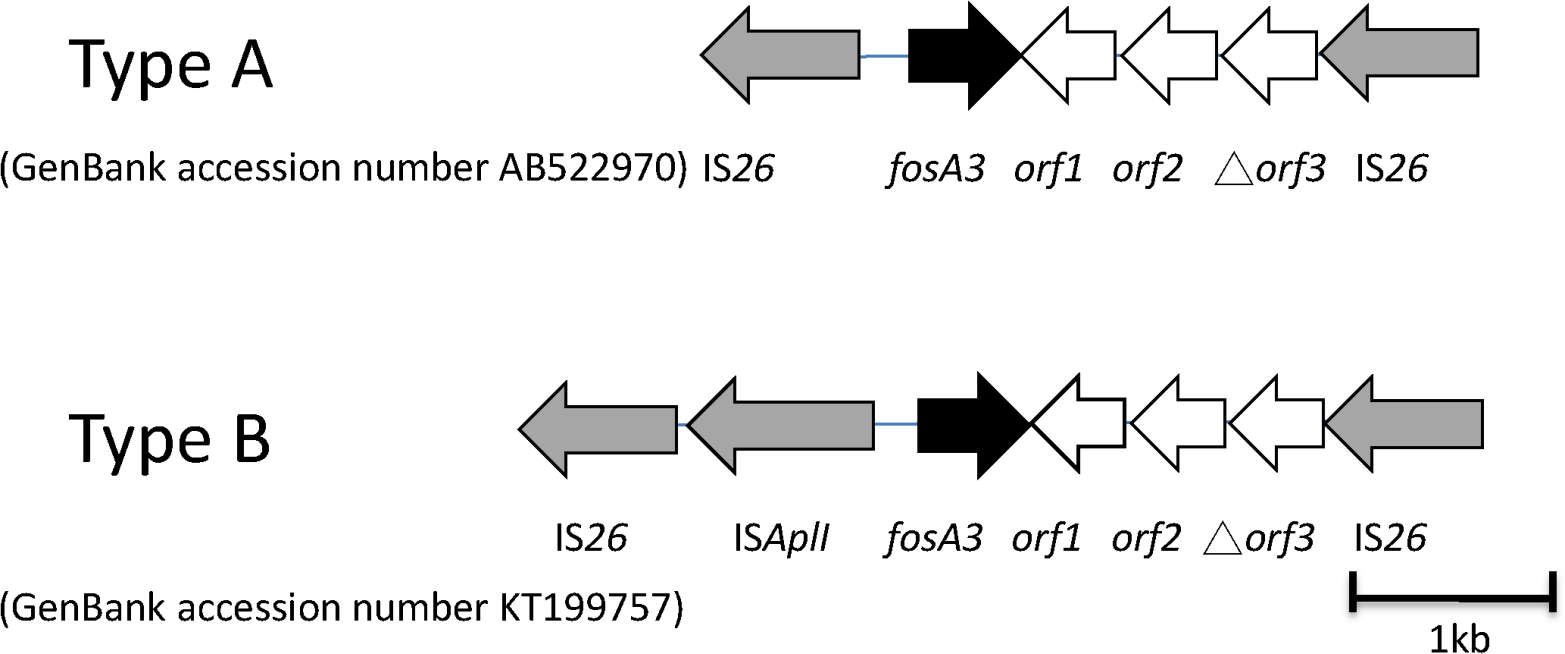
Representation of the sequences flanking *fosA3*. Genes are shown as arrows with the direction of transcription indicated by the arrowheads.

**Fig 3:**
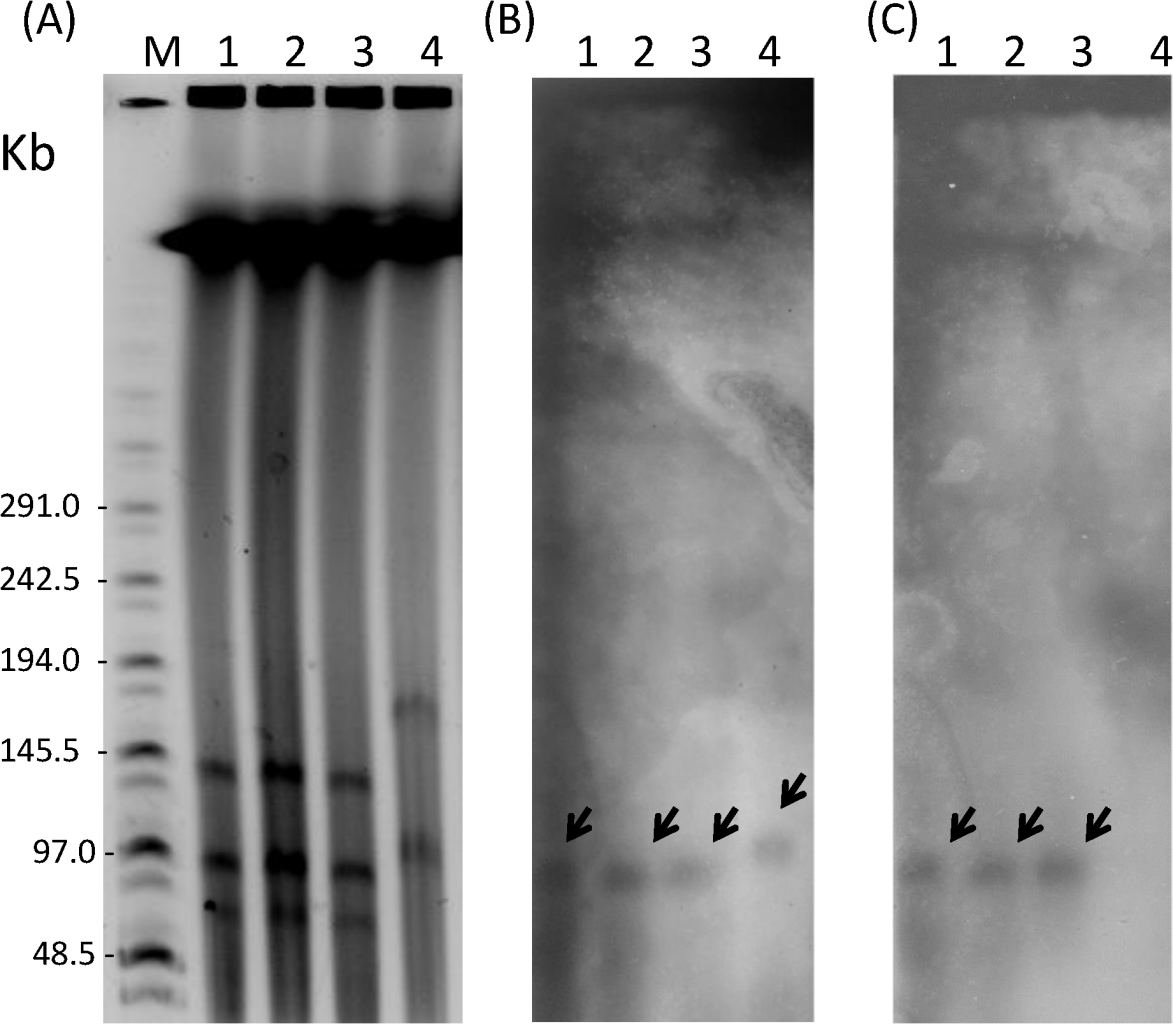
PFGE analysis of *fosA3*-producing *E*. *coli*. (A) S1-nuclease digested plasmid profiles separated by PFGE. (B) S1-nuclease digested plasmid profiles hybridized with a *fosA3* probe. The arrows showed the location of *fosA3* genes. (C) S1-nuclease digested plasmid profiles hybridized with a *bla*_CTX-M-group 9_ probe. The arrows showed the location of *bla*_CTX-M-group 9_ genes. Lane M, MidRange II PFG Marker; lane1, A10; lane 2, A11; lane 3, A12 and lane 4, K66.

For the four *fosA3*-habouring isolates (A10, A11, A12, and K66), the surrounding region of *fosA3* was flanked by two IS*26* elements (type A; Fig 2). Three isolates (A10 from farm A, A12 from farm C, and K66 from KMUH) showed a 100% identity to the GenBank accession number AB522970, which contained *fosA3*, *orf1*, *orf2* and truncated *orf3* (type A; Fig 2) [14]. The other isolate (A11 from farm A) had an IS*AplI* inserting a similar structure between IS*26* and *fosA3* (type B; Fig 2) (GenBank accession number was KT199757). The PFGE and MLST typing revealed that 3 different pig isolates (pulsotype XXXIV and ST744) were the same genotype, which differed from the human isolate (pulsotype XXX and ST2310) (Table 3). The plasmid profiles of the pig isolates were similar (146, 101, and 85 kbp) whereas the human isolate had 2 different sized plasmids (184 and 113 kbp). The locations of the *fosA3* and *bla*_CTX-M-group 9_ genes were analyzed by S1 nuclease-PFGE and hybridization with specific probes for the *fosA3* and *bla*_CTX-M-group 9_ genes, respectively. The *fosA3* gene was hybridized with a 101 kbp IncN-type plasmid in the pig isolates and a 113 kb IncB/O-type plasmid in the human isolate (Fig 3B and Table 3). In addition, the 101 kbp IncN-type plasmid was hybridized with the *bla*_CTX-M-group 9_ probe in the pig isolates (Fig 3C), indicating the co-localization of the *fosA3* and *bla*_CTX-M-group 9_ genes in the pig isolates. Although, IncB/O-type plasmid contained *fosA3* without *bla*_CTX-M-group 9_ gene, *bla*_CMY-2_ was found in transformant of IncB/O-type plasmid (K66) by PCR detection. The antibiotic susceptibility tests revealed that these 4 clinical isolates were resistant to ampicillin, amikacin, tetracycline, levofloxacin, chloramphenicol, fosfomycin, and cefotaxime without meropenem, while the transformants were only resistant to ampicillin, cefotaxime and fosfomycin.

**Table 3:** Characterization of 4 *fosA3*-habouring isolates and their transformants.

Strain	Sources	PFGE type	MLST	Plasmid		Resistant gene content^b^	Environment of *fosA3* ^c^	Resistant profiles^a^	
				replicon	sizes (kb)			parental	transformant
**A10**	Pig feces (Farm A)	XXIV	744	IncN, IncFIB IncFrepB, IncP	146, 101, 85	*bla*_CTX-M-group 9_, *fosA3*	Type A	AM, AMK, TC, LVX, CHL, FOS, CTX	AM, FOS, CTX
**A11**	Pig feces (Farm A)	XXIV	744	IncN, IncFIB IncFrepB, IncP	146, 101, 85	*bla*_CTX-M-group 9_, *fosA3*	Type B	AM, AMK, TC, LVX, CHL, FOS, CTX	AM, FOS, CTX
**A12**	Pig feces (Farm C)	XXIV	744	IncN, IncFIB IncFrepB, IncP	142, 101, 85	*bla*_CTX-M-group 9_, *fosA3*	Type A	AM, AMK, TC, LVX, CHL, FOS, CTX	AM, FOS, CTX
**K66**	Urine (KMUH)	XXX	2310	IncI1, IncFIB IncFrepB IncB/O, IncP	184, 113	*bla*_CTX-M-group 9_, CMY-2, *fosA3*	Type A	AM, AMK, TC, LVX, CHL, FOS, CTX	AM, FOS, CTX

a. Antibiotic susceptibility test were included AM, AMK, TC, LVX, CHL, FOS, CTX, and MEM. AM = Ampicillin, AMK = Amikacin, TC = Tetracycline, LVX = levofloxacin, CHL = Chloramphenicol, FOS = Fosfomycin, CTX = cefotaxime and, MEM = Meropenem.

b. The transferring plasmid containing resistant gene was underlined.

c. Structure of Type A and B were described in Fig 2.

### Modification of the fosfomycin target MurA

Amino acid substitutions of MurA were found in two pig isolates: A12 (Asn67Ile), A19 (Phe151Ser and Trp164Ser), and one hospital isolate: K55 (Val146Ala and His159Tyr), respectively. To test the correlation between these amino acid substitutions and fosfomycin resistance, wild type and mutant MurA were constructed and expressed in *E*. *coli* DH5α (Table 4). The variations of MurA were shown to cause a 16-fold increase of the fosfomycin MIC compared to the wild-type MurA (Table 4). These results indicate that the amino acid substitutions in MurA were related to fosfomycin resistance.

**Table 4:** Evaluation of fosfomycin susceptibility in overexpressing wild type and mutant MurA.

Bacterial strain	Enzyme overexpressed	MIC (μg/ml)^a^
DH5α	None	0.25
DH5α/*murA* (25922)	MurA (wild-type)	8
DH5α/*murA* (A12)	MurA (Asn67Ile)	128
DH5α/*murA* (A19)	MurA (Phe151Ser and Trp164Ser)	128
DH5α/*murA* (K55)	MurA (Val146Ala and His159Tyr)	128

MIC, minimal inhibitory concentration.

^a^ MICs were determined in the presence of 1mM Isopropyl β-D-1-thiogalactopyranoside (IPTG) to induce MurA expression.

## Discussion

To our knowledge, this study was the first to investigate fosfomycin resistance of both human and pig ESBL-producing *E*. *coli* isolates in Taiwan. Thirteen (9%) ESBL-producing *E*. *coli* isolates were resistant to fosfomycin in the study. The resistant rate to fosfomycin was lower in human isolates (6% in KMUH and 11% in PTH) than the resistance rate in pig isolates (22%). Among the only two studies on fosfomycin susceptibility from human isolates in Taiwan, one study revealed that 95.5% ESBL-producing *E*. *coli* isolates were susceptible to fosfomycin [23] and the other revealed that 100% *E*. *coli* isolates were susceptible to fosfomycin [24]. According to the two reports, fosfomycin is a therapeutic choice for treating ESBL-producing *E*. *coli* infection in Taiwan. We identified a relatively high fosfomycin resistant rate for pig ESBL-producing *E*. *coli* isolates. Our results suggested antibiotic selection pressure might exist in pig farms. The molecular epidemiology analysis by the PFGE found no clonal associations between human and pig isolates. Clonal spread of three pulsotypes might occur in two hospitals.

In this study, four isolates contained the *fosA3* gene in a plasmid, and nine isolates displayed the mutation in *murA*, *glpT*, *uhpT*, *uhpA*, *ptsI*, and *cyaA* genes. Nine and eight isolates did not grow on minimal medium agar supplemented with G3P and G6P, respectively. This result indicated that the UhpT and/or GlpT transporter may be defective in these isolates. Although, amino acids substitutions in transporters or regulated genes were not evidenced by *in vitro* experiments in this study, functionless transporters (GlpT or UhpT) could be detected by minimal medium agar with G3P or G6P, as the previously reported [21,22]. Takahata et al. reported 6 fosfomycin resistant clinical *E*. *coli* isolates that harbored different fosfomycin-related gene mutations [22]. The *glpT* gene mutation (4 isolates) and the loss of the entire *uhpT* gene (2 isolates) decreased the fosfomycin uptake into the bacterial cells, leading to fosfomycin resistance. The other study by Nilsson et al. investigated fosfomycin resistance mechanisms in 13 clinical *E*. *coli* isolates [21]. A mutation in the *cyaA* (5 isolates) and *ptsI* (2 isolates) genes impaired both *glpT* and *uhpT* expression. In our study, three and one isolates carried mutations in their *uhpT* and *glpT* genes, respectively. MurA has been reported to confer clinical fosfomycin resistance at a low fitness cost [25]. The other study described two isolates that contained the *murA* mutation (Asp369Asn or Leu370Ile), leading to fosfomycin resistance [22]. Our results revealed new variations (Asn67Ile, Phe151Ser and Trp164Ser, Val146Ala and His159Tyr, respectively) in MurA (Table 4) that might affect fosfomycin binding to the Cys-115 residue (active site) or the three conserved positively charged residues (Lys22, Arg120 and Arg397) in MurA [26].

In East Asia, most countries have found plasmid-mediated *fosA3* in ESBL-producing *E*. *coli* isolates [8,9,11–13]. Ho et al. found *fosA3* and *bla*_CTX-M_ genes were coharboured on conjugative plasmids with F2:A-:B- (n = 2), N (n = 1), F-:A-:B1 and N (n = 1) and untypable (n = 2) replicons in Hong Kong [8]. Hou et al. reported *fosA3* and *bla*_CTX-M_ genes were coharboured on conjugative plasmids with F33:A-:B- (n = 2), F2:A-:B- (n = 1), and N (n = 4) in China [11]. Lee et al. reported *fosA3* and *bla*_CTX-M_ genes were coharboured on conjugative plasmids with FII (n = 3), and N (n = 1) in Koera [12]. SaTo et al. reported *fosA3* and *bla*_CTX-M_ genes were coharboured on conjugative plasmids with I1 (n = 2), N (n = 1) and FII (n = 2) in Japan [13]. We reported the first detection of *E*. *coli* isolates containing an IncN-type plasmid (101 kbp) carrying *fosA3* and *bla*_CTX-M-G9_ genes from pig feces and an IncB/O-type plasmid (113 kbp) carrying *fosA3* from a human urine specimen in Taiwan (Table 3). In literature, the IncN-type plasmid carries different important resistance determinants such as CTX-M, VIM-1, KPC-2 and NDM-1 [27,28]. The other IncB/O-type plasmid (113 kbp) that contains *fosA3* and *bla*_CMY-2_ from the human urine specimen was similar to the pX6SA plasmid (120 kbp) harboring IncB/O replicon and *fosA3*, which was reported from cattle fecal specimens in Hong Kong [9].

Research regarding the transposable elements surrounding ESBLs was limited in Taiwan [29,30]. The detection of ESBLs and the linkage of transposable elements were investigated in this study. The most common ESBLs were CTX-M-group 1, CTX-M-group 9 and CMY-2 in *E*. *coli* in Taiwan. Our results revealed the same transposable elements surrounding ESBLs in human and pig isolates that indicate that ESBLs could be transferred via a plasmid or transposon between human and pig hosts. SHV was predominant in ESBL-producing *K*. *pneumoniae* isolates in Taiwan [30] whereas a low percentage (5.5%) of SHV was found in the ESBL-producing *E*. *coli* isolates in this study (Table 2). IS*26* was related to disseminated antibiotic resistance genes worldwide[31, 32]. In this study, IS*26*, which was located upstream of *bla*SHV, and *fosA3*, was associated with the dissemination of antibiotic resistance genes in Taiwan (S1 Fig and Fig 2). IS*Ecp1* was mostly found upstream of *bla*CTX-M in Spanish, French, Indian and Turkish [18,33,34]. Our study identified the role of IS*Ecp1* in Taiwan that supported its role for global *bla*CTX-M dissemination. Besides, IS*Ecp1* was upstream of the *bla*CTX-M-group 1 and *bla*CTX-M-group 9 genes with different spacer regions, (S1 Fig). Our previous study demonstrated that *bla*CTX-M expression was correlated with different spacer sequences (42 and 127 bp), which affected *bla*CTX-M expression [35]. In this study, the 42 bp spacer region was found predominant (S1 Table).

It should be noted that the number of pig isolates was small in this study. The prevalence of resistance to fosfomycin required further studies with a larger isolate number. Due to the limited sample size, the clonal relationship between human and pig isolates required further studies with a larger isolate number.

In conclusion, the susceptibility rates of fosfomycin were different between human and pig ESBL-producing *E*. *coli* isolates. The major resistance mechanism of fosfomycin was amino acid variation in chromosomally encoded fosfomycin-related genes such as the antibiotic target gene (*murA*), GlpT and UhpT transporters (*glpT* and *uhpT*) and regulating genes (*uhpA*, *cyaA*, and *ptsI*). The IncN-type plasmid carrying *fosA3* and *bla*_CTX-M-group 9_ in pig isolates is of concern. In addition, the horizontal *fosA3*-transferring via the IS*26* mobile element between human and pig isolates might exist in Taiwan and warrant further monitoring.

## Supporting Information

S1 Fig Representation of the transposable elements surrounding *bla*CTX-M and *bla*SHV genes. The transcriptional mobile elements and space region were detected by PCR and sequencing.
(TIFF)

S1 Table ESBLs and linkage of transposable elements in *E*. *coli* isolates. (DOCX)
