# Supplementary figures and images for: Characterization of Fosfomycin Resistant Extended-Spectrum β-Lactamase-Producing Escherichia coli Isolates from Human and Pig in Taiwan
Source: PLoS One. 2015 Aug 17;10(8):e0135864. doi: 10.1371/journal.pone.0135864 (PMC4539220; doi:10.1371/journal.pone.0135864)

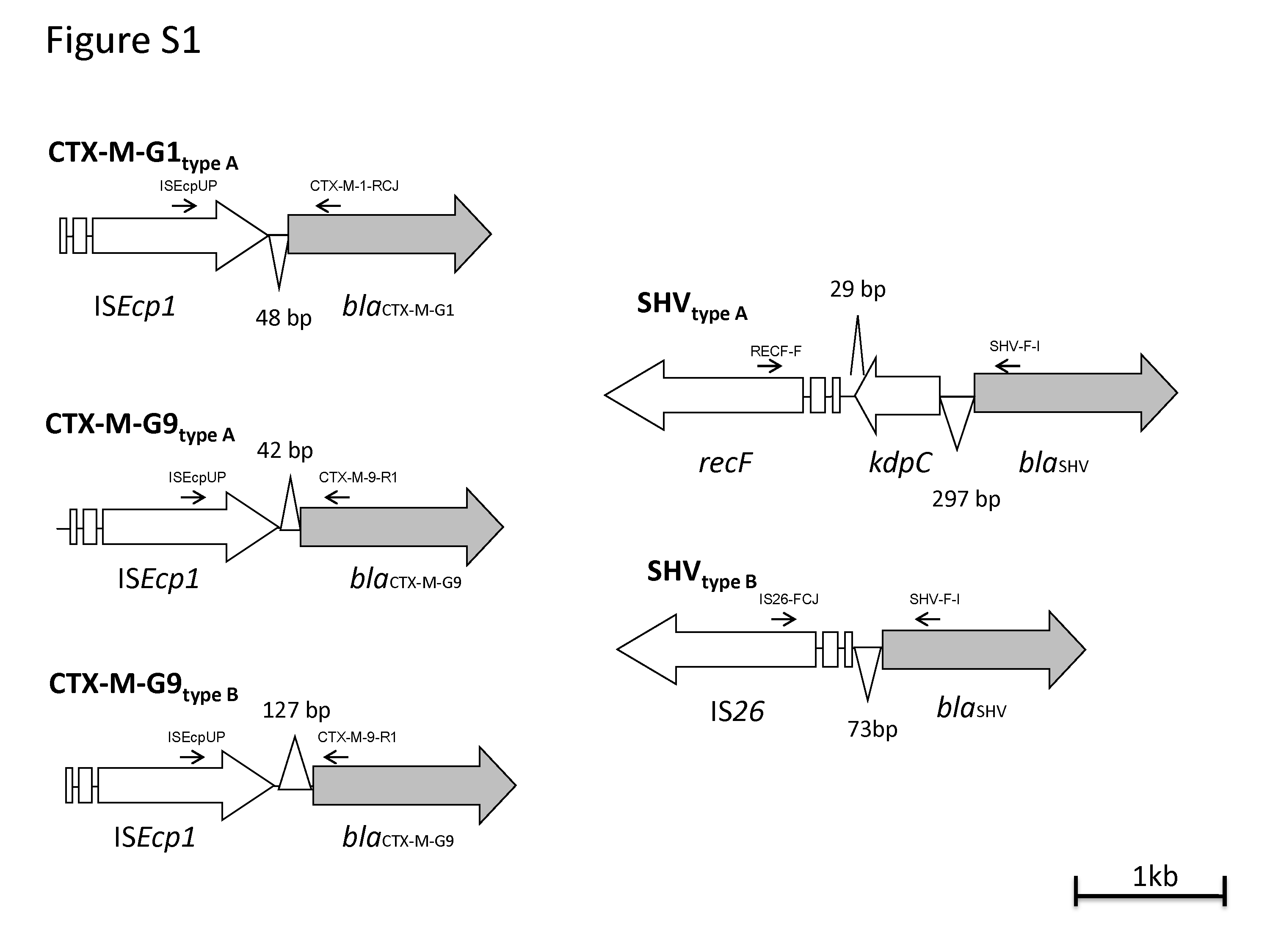

Supplement: S1 Fig — The transcriptional mobile elements and space region were detected by PCR and sequencing. (TIFF) [file pone.0135864.s001.tiff]
