# Supplementary material for: Characterization of Fosfomycin Resistant Extended-Spectrum β-Lactamase-Producing Escherichia coli Isolates from Human and Pig in Taiwan
Source: PLoS One. 2015 Aug 17;10(8):e0135864. doi: 10.1371/journal.pone.0135864 (PMC4539220; doi:10.1371/journal.pone.0135864)
Supplement: S1 Table — (DOCX) [file pone.0135864.s002.docx]

**Table S1.** ESBLs and linkage of transposable elements in *E. coli* isolates

| *bla*_SHV_ | SHV_Type A_ (%) | SHV_TypeB_ (%) |  |  |
| --- | --- | --- | --- | --- |
| KMUH | 4/8 (50) | 4/8 (50) |  | 8/8 (100) |
| PTH | 0/1 (0) | 0/1 (0) |  | 0/1 (0) |
| Pig isolates | 0/0 (0) | 0/0 (0) |  | 0/0 (0) |
| *bla*_CTX-M-group 1_ | CTX-M-G1_Type A_ (%) |  |  |  |
| KMUH | 15/18 (83) |  |  | 15/18 (83) |
| PTH | 9/10 (90) |  |  | 9/10 (90) |
| Pig isolates | 3/6 (50) |  |  | 3/6 (50) |
| *bla*_CTX-M-group 9_ | CTX-M-G9_Type A_ (%) | CTX-M-G9_Type B_ (%) |  |  |
| KMUH | 19/36 (52) | 15/36 (41) |  | 34/36 (94) |
| PTH | 3/7 (42) | 0/7 (0) |  | 3/7 (42) |
| Pig isolates | 5/14 (35) | 3/14 (28) |  | 8/14 (57) |
